# Lurasidone for the treatment of depressive symptoms in schizophrenia: analysis of 4 pooled, 6-week, placebo-controlled studies

**DOI:** 10.1017/S1092852914000285

**Published:** 2014-06-23

**Authors:** Henry A. Nasrallah, Josephine B. Cucchiaro, Yongcai Mao, Andrei A. Pikalov, Antony D. Loebel

**Affiliations:** 1 Department of Neurology and Psychiatry, St. Louis University, St. Louis, Missouri, USA; 2 Sunovion Pharmaceuticals Inc., Fort Lee, New Jersey, USA

**Keywords:** Atypical antipsychotic, depression, depressive symptom, lurasidone, schizophrenia

## Abstract

**Objective:**

Depressive symptoms are common in schizophrenia and can worsen outcomes and increase suicide risk. Lurasidone is an atypical antipsychotic agent indicated for the treatment of schizophrenia and for the treatment of major depressive episodes associated with bipolar I disorder. This post hoc analysis evaluated the effect of lurasidone on depressive symptoms in patients with schizophrenia.

**Methods:**

Patient-level data were pooled from 4 similarly designed, double-blind, placebo-controlled, 6-week registration studies of lurasidone (40–160 mg/d) in adult patients with an acute exacerbation of schizophrenia. Changes in depressive symptoms, measured by the Montgomery–Åsberg Depression Rating Scale (MADRS), were analyzed for the overall sample and for subgroups of patients stratified by baseline MADRS scores.

**Results:**

MADRS assessments at baseline and endpoint (day 42 or last observation carried forward [LOCF]) were available for 1330 patients. Patients receiving lurasidone experienced significantly greater decreases in MADRS score (–2.8, least-squares [LS] mean change, LOCF) compared with patients receiving placebo (–1.4, *P* < .001, effect size 0.24). Analysis of change in MADRS score (LOCF) by baseline symptom severity (MADRS score of ≥12, ≥14, ≥16, ≥18) showed significantly greater improvement for lurasidone-treated patients across all severity groups; effect sizes ranged from 0.25 to 0.34. Among patients with a baseline MADRS score of ≥12, depressive symptom remission (defined as MADRS score <10 at LOCF endpoint) was attained by 45.0% of lurasidone-treated patients and 36.3% of patients receiving placebo (*P* < .05).

**Conclusions:**

In a pooled analysis of short-term, placebo-controlled studies, lurasidone significantly improved depressive symptoms in patients with schizophrenia.

## Clinical Implications

• Depressive symptoms are common in schizophrenia, and their presence can worsen clinical outcomes.
• This pooled analysis of patient-level data from 4 randomized, placebo-controlled, 6-week studies in patients with schizophrenia found that treatment with lurasidone resulted in significantly greater improvement in depressive symptoms compared with placebo.
• Lurasidone may provide a useful therapeutic option for patients with depressive symptoms associated with schizophrenia.

## Introduction

Schizophrenia is a chronic, debilitating neuropsychiatric disorder characterized by hallucinations, delusions, disorganized thoughts, and bizarre behavior.^1^ The worldwide prevalence of schizophrenia is estimated to be between 0.4% and 1.1% of the adult population,^2^ ^,^ ^3^ and the prevalence of clinically significant depressive symptoms in patients with schizophrenia has been estimated at between 25% and 50%.^4^ ^–^ ^8^ Depressive symptoms have long been regarded as integral to the psychopathology associated with schizophrenia.^9^ The presence of depressive symptoms in patients with schizophrenia reduces patients’ ability to function, increases the burden imposed by the underlying psychotic illness, and worsens outcomes and prognosis.^5^ ^,^ ^10^ ^–^ ^14^

Lurasidone is an atypical antipsychotic agent with high affinity for dopamine D_2_, 5-hydroxytryptamine (5-HT)_2A_, and 5-HT_7_ receptors (antagonist effect), and moderate affinity for 5-HT_1A_ receptors (partial agonist effect).^15^ Multiple studies have demonstrated the efficacy of lurasidone in the treatment of schizophrenia,^16^ ^–^ ^22^ and lurasidone has shown antidepressant-like activity in animal models of depression.^15^ ^,^ ^23^ A recent study in 5HT_7_ knock-out mice indicated that the antidepressant effect of lurasidone requires the presence of the 5HT_7_ receptor.^23^ Thus, both the receptor-binding profile and preclinical data for lurasidone indicate its potential for ameliorating depressive symptoms. Lurasidone was recently approved in the United States for the treatment of major depressive episodes associated with bipolar I disorder, both as monotherapy and as adjunctive therapy with lithium or valproate, on the basis of 2 pivotal trials.^24^ ^,^ ^25^ The objective of the present post hoc analysis was to evaluate the effect of lurasidone on depressive symptoms in patients with schizophrenia.

## Methods

Four similarly designed, placebo-controlled studies of lurasidone were identified that included the Montgomery–Åsberg Depression Rating Scale (MADRS) for evaluation of changes in depressive symptoms in patients with schizophrenia (Table 1). Patient-level data from these studies were pooled in the current analysis. Each study (1 phase II study conducted in the U.S. and 3 multiregional phase III studies) examined the efficacy and safety of lurasidone in the treatment of patients experiencing an acute exacerbation of schizophrenia; the primary results of all 4 studies have been reported elsewhere.^17^ ^–^ ^20^

**Table 1:** Summary of studies^*a*^ included in this pooled analysis

Study number	Dates, locations	Study medication^*b*^	Patients (n) by study
Study 1 (D1050196)^17^	May 2004–Dec 2004,	Lurasidone 80 mg/d	86
Phase II	22 U.S. sites	Placebo	83
Study 2 (D1050229)^18^	Oct 2007–Dec 2008,	Lurasidone 40 mg/d	120
Phase III	multinational,	Lurasidone 80 mg/d	116
	48 sites (21 U.S. sites)	Lurasidone 120 mg/d	123
		Placebo	122
Study 3 (D1050231)^19^	Jan 2008–June 2009,	Lurasidone 40 mg/d	116
Phase III	multinational,	Lurasidone 120 mg/d	109
	52 sites (25 U.S. sites)	Placebo	111
		Olanzapine 15 mg/d^*c*^	119
Study 4 (D1050233)^20^	Oct 2008–June 2010,	Lurasidone 80 mg/d	116
Phase III	multinational,	Lurasidone 160 mg/d	112
	63 sites (24 U.S. sites)	Placebo	116
		Quetiapine XR 600 mg/d^*c*^	110

a All were 6-week, randomized, placebo-controlled, parallel-group, fixed-dose studies.

b Study medication was administered in the morning with food in Studies 1-3, and in the evening with food in Study 4.

c Included in the individual study to confirm assay sensitivity, but not included in this pooled analysis.

The conduct of each study was consistent with the Declaration of Helsinki and Good Clinical Practice guidelines. Each study was approved by an institutional review board or independent ethics committee, and all patients provided written informed consent prior to study enrollment.

### Patients

Inclusion/exclusion criteria were similar across the 4 studies.^17^ ^–^ ^20^ Key inclusion criteria were as follows: adults aged 18–75 years with a diagnosis of schizophrenia (according to the *Diagnostic and Statistical Manual of Mental Disorders*, fourth edition [DSM-IV]) for at least 1 year who were experiencing an acute exacerbation of psychotic symptoms, as reflected by a Clinical Global Impression of Severity (CGI-S) score of ≥4 (moderate or greater) and a Positive and Negative Syndrome Scale (PANSS) total score of ≥80, including a score of ≥4 (moderate) on 2 or more of the following 5 PANSS items: delusions, conceptual disorganization, hallucinations, unusual thought content, and suspiciousness.^*^

Patients were excluded from study participation if they had a DSM-IV diagnosis of schizoaffective disorder; an acute or unstable medical condition; evidence of any other chronic disease of the central nervous system; alcohol or other drug abuse/dependence within the past 3–6 months; evidence of a severe, chronic movement disorder; or if they were judged by the study investigator to be at risk for suicide.

### Study design

Patients were tapered off psychotropic medications prior to a 3- to 7-day, single-blind, placebo run-in period. Patients receiving treatment with antidepressants were required to discontinue such medications at least 1 week prior to study entry, with time since discontinuation extended to at least 3 weeks for those taking monoamine oxidase inhibitors and at least 1 month for those taking fluoxetine (because of the long half-life).^†^ Concomitant administration of antidepressant medication was prohibited for the duration of the study. Limited use of lorazepam, temazepam, and zolpidem (for anxiety/agitation or insomnia) and benztropine, biperiden, trihexyphenidyl, or diphenhydramine (for extrapyramidal symptoms) was permitted on an as-needed basis, but not within 8 hours of MADRS assessments.

Patients who continued to meet entry criteria after the single-blind placebo run-in period were randomized to receive placebo, fixed-dose lurasidone, or an active comparator for 6 weeks (Table 1). Depending on the study, the daily dose of lurasidone was 40 mg, 80 mg, 120 mg, or 160 mg. Study medication was taken in the morning, with food, in 3 of the 4 studies and in the evening, with food, in 1 study (Table 1). All patients were initially hospitalized for a minimum of 3–4 weeks of treatment; hospital discharge was then permitted for patients who had achieved a CGI-S score of ⩽3 and were judged by the investigator to be clinically stable.^‡^

The severity of depressive symptoms was determined using the 10-item MADRS,^26^ scored by trained raters during clinical interviews. In all studies, the MADRS was administered at baseline (prior to the first dose of study medication) and at endpoint, defined as day 42 or the last observation available.

### Statistical analysis

Patient-level data from the 4 studies were pooled for patients receiving lurasidone (40 mg/d, 80 mg/d, 120 mg/d, or 160 mg/d) or placebo. The analyzed population included all patients with MADRS scores at baseline and at least 1 post-baseline assessment. Efficacy analyses of lurasidone versus placebo were performed using an analysis of covariance (ANCOVA), with change in score from baseline to endpoint (last observation carried forward [LOCF]) as the dependent variable; treatment group, study, and site within study as main effects; and baseline MADRS score as a covariate. Because the MADRS was included as a secondary assessment in these studies, analyses involving the MADRS were not adjusted for multiple comparisons. Effect sizes were calculated from the ANCOVA analysis (LOCF) as the between-treatment group difference in least-squares (LS) mean change scores (the absolute value of lurasidone minus placebo) divided by the pooled standard deviation of the change scores.

Treatment response was defined as a 50% reduction in MADRS score from baseline, and remission was defined as a final MADRS score of <10.^27^ Response and remission rates at endpoint (LOCF) were determined for lurasidone (dose groups pooled) and placebo, and the number needed to treat (NNT) was calculated for both outcomes as the reciprocal of the absolute difference in the rates for lurasidone and placebo (eg, 1/[response rate with lurasidone minus response rate with placebo], rounded up to the next higher whole number).^28^ Differences between lurasidone (dose groups pooled) and placebo in the response rate and the remission rate were analyzed using logistic regression models, with treatment, study, and baseline value as explanatory variables.

The correlation between change from baseline to LOCF endpoint in MADRS total score and PANSS total score was assessed utilizing Pearson product moment correlation coefficients (*r*), which were computed separately for the placebo group and the lurasidone group (doses pooled). The proportion of variance shared by the two variables was evaluated using *r* ^*2*^.

## Results

MADRS total scores at baseline and post-baseline were available for 1330 patients with acute schizophrenia (N = 898 lurasidone, N = 432 placebo), who were included in the pooled analysis. Overall baseline demographic and clinical characteristics were similar for patients who were treated with lurasidone (dose groups pooled) and patients who received placebo (Table 2). In these fixed-dose studies, 26.3% of patients received lurasidone 40 mg/d, 35.4% received 80 mg/d, 25.8% received 120 mg/d, and 12.5% received 160 mg/d.

**Table 2:** Demographic and baseline clinical characteristics

Characteristic	Lurasidone (N = 898)	Placebo (N = 432)
Age (y), mean (SD)	38.3 (10.9)	38.3 (10.6)
Men, n (%)	649 (72.3)	311 (72.0)
Race, n (%)		
White	424 (47.2)	189 (43.8)
Black	289 (32.2)	151 (35.0)
Asian	154 (17.1)	68 (15.7)
Other	31 (3.5)	24 (5.6)
Age of onset of illness (y), mean (SD)^*a*^	24.4 (8.4)	24.5 (8.1)
Prior hospitalizations ≥4, n (%)^*a*^	445 (49.6)	183 (42.4)
PANSS total score, mean (SD)	96.7 (10.8)	96.5 (10.9)
CGI-S score, mean (SD)	4.9 (0.6)	4.9 (0.6)
MADRS score		
Mean (SD)	11.4 (7.2)	11.9 (7.1)
Median (range)	10 (0–40)	11 (0–46)

^*a*^Data not collected for Study 1.

CGI-S: Clinical Global Impression of Severity; MADRS: Montgomery–Åsberg Depression Rating Scale; PANSS: Positive and Negative Syndrome Scale; SD: standard deviation.

**Figure 1:**
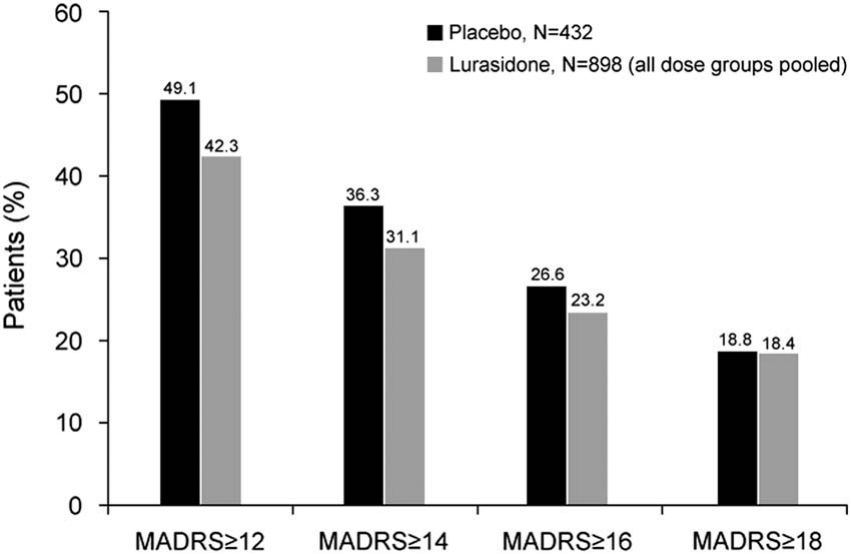
Proportion of patients with Montgomery–Åsberg Depression Rating Scale (MADRS) scores above selected cutoff values at study baseline.

At baseline, MADRS total scores ranged from 0 to 46, with a mean score of 11.4 for the lurasidone group (doses pooled) and 11.9 for the placebo group. Figure 1 shows the proportion of patients with a baseline MADRS score of ≥12, ≥14, ≥16, and ≥18. Mean baseline score on PANSS item G6 (depression) was 2.6 for both groups, indicating the presence of mild depressive symptoms in this patient population at study baseline.

**Figure 2:**
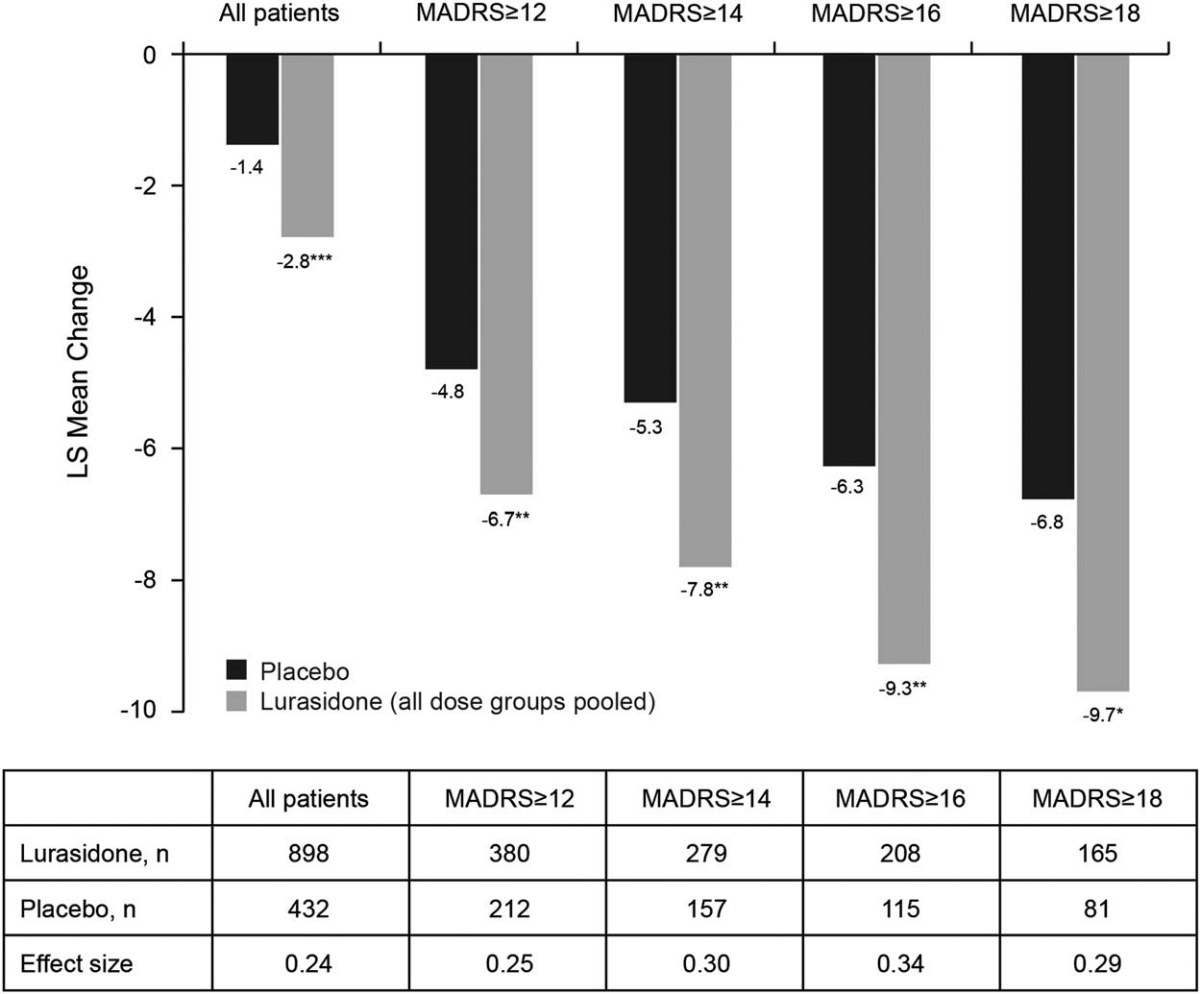
Least-squares (LS) mean change in Montgomery–Åsberg Depression Rating Scale (MADRS) scores at endpoint (last observation carried forward) for all patients and by baseline MADRS score. ^*^ *P* < .05, ^**^ *P* < .01, ^***^ *P* < .001 for lurasidone compared with placebo.

At endpoint (week 6, LOCF), the mean MADRS total score was 8.4 for the lurasidone group (doses pooled) compared with 10.2 for the placebo group. A significantly greater decrease in MADRS total score (LOCF) was observed for patients treated with lurasidone (LS mean change, –2.8) compared with patients receiving placebo (–1.4, *P* < .001; Figure 2). The LS mean decrease (LOCF) in PANSS item G6 (depression) score was also significantly greater with lurasidone (–0.6) than with placebo (–0.4; *P* < .01). An observed case analysis of patients who completed 6 weeks of treatment and had a MADRS assessment at day 42 (N = 679 lurasidone, N = 296 placebo) showed similar results: LS mean change in MADRS total score from baseline was –4.2 for the lurasidone group and –3.0 for the placebo group (*P* < .01).

Analysis of change in MADRS total score (LOCF) stratified by baseline severity of depression (MADRS total score of ≥12, ≥14, ≥16, ≥18) showed significantly greater improvement for lurasidone-treated patients in all baseline severity subgroups; effect size was 0.24 for all patients and ranged from 0.25 to 0.34 across the patient subgroups defined by baseline MADRS severity (Figure 2). The proportion of patients who were treatment responders with respect to depressive symptoms (≥50% reduction in MADRS total score) was numerically greater with lurasidone than placebo at all levels of baseline depression severity; however, differences between lurasidone and placebo in the rate of treatment response were not statistically significant. The corresponding NNTs for lurasidone response ranged from 11 to 14 (Figure 3A). Remission (defined as a MADRS total score of <10 at endpoint [LOCF]) was attained by a greater proportion of patients treated with lurasidone compared with placebo. Among the MADRS-defined baseline severity groups (score of ≥12, ≥14, ≥16, ≥18), the difference in remission rate for lurasidone versus placebo was statistically significant only for baseline MADRS ≥12 (*P* < .05). NNTs for remission with lurasidone ranged from 11 to 13 (Figure 3B).

**Figure 3a:**
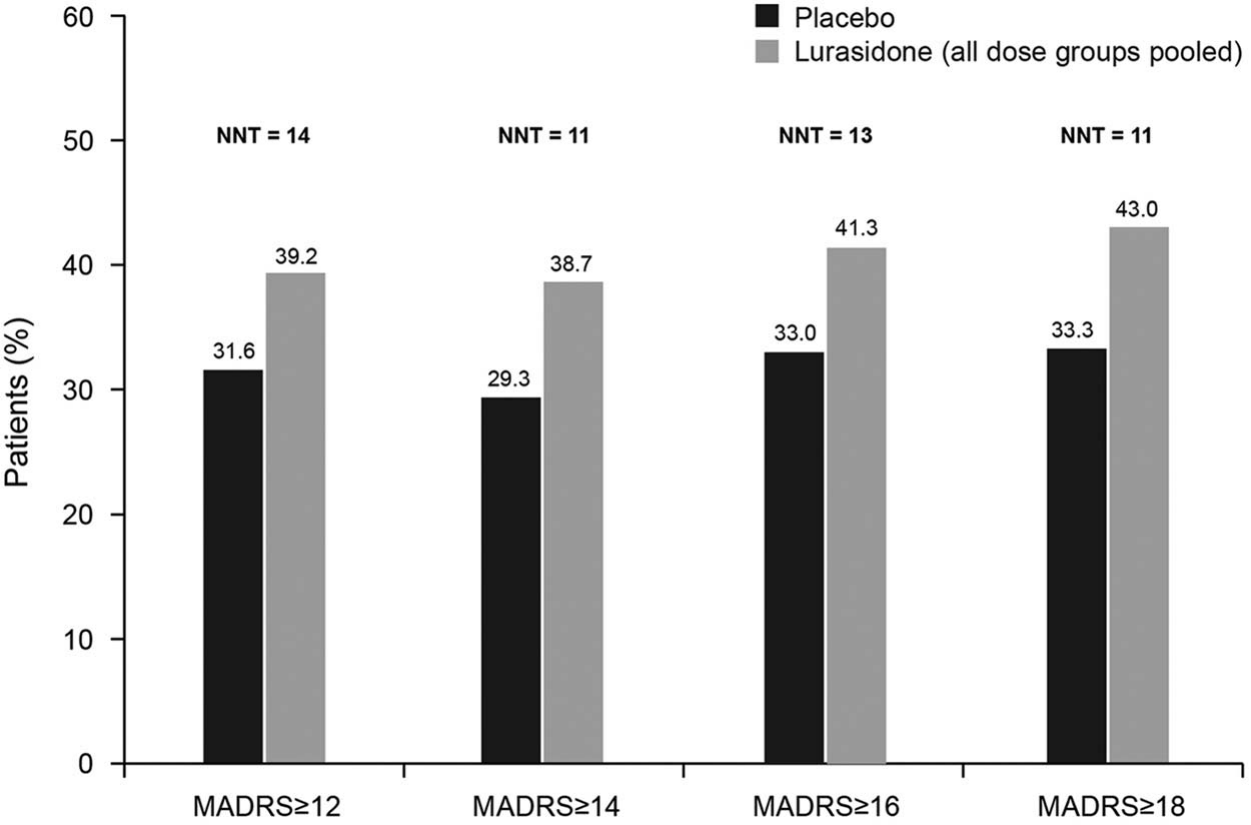
Proportion of patients who were depressive symptom responders at endpoint (last observation carried forward), by severity of depressive symptoms at baseline. (Depressive symptom responders are defined as those with a ≥50% decrease from baseline in Montgomery–Åsberg Depression Rating Scale [MADRS] score.) *P* values for comparisons of lurasidone (dose groups pooled) versus placebo were not significant. NNT: number needed to treat.

**Figure 3b:**
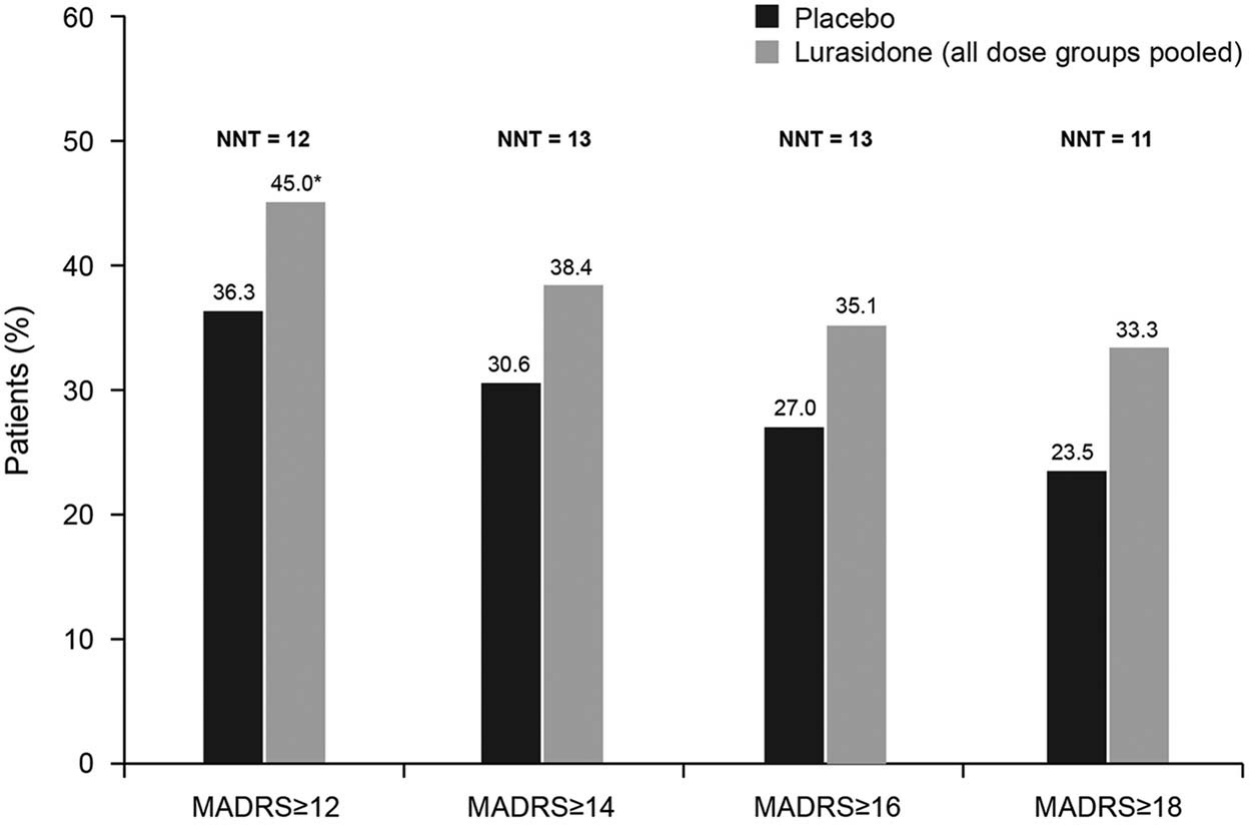
Proportion of patients who were depressive symptom remitters at endpoint (last observation carried forward), by severity of depressive symptoms at baseline. (Depressive symptom remitters defined as those with a ≥50% decrease from baseline in Montgomery–Åsberg Depression Rating Scale [MADRS] score.) ^*^ *P* < .05 for lurasidone (dose groups pooled) versus placebo. NNT: number needed to treat.

The changes in MADRS total score were further analyzed by fixed lurasidone dose assignment in the respective studies. When compared with placebo, significantly greater reduction in depressive symptoms, as indicated by improvement in MADRS total score at endpoint (LOCF), was demonstrated for lurasidone 80 mg/d (*P* < .001) and lurasidone 160 mg/d (*P* < .001), but not lurasidone 40 mg/d (*P* = 0.22) or 120 mg/d (*P* = 0.95).

The correlation of change in MADRS total score and change in PANSS total score, from baseline to LOCF endpoint, was 0.46 in the lurasidone group (doses pooled) and 0.49 in the placebo group; both *P* < .001. The proportion of shared variance between the 2 variables is the square of the correlation; thus, change in PANSS total score accounted for 21% (pooled lurasidone group) to 24% (placebo group) of the variance in the MADRS change score.

Detailed safety and tolerability findings for each of the 4 studies included in this analysis have been previously published.^17^ ^–^ ^20^ The most common adverse events with lurasidone in short-term studies of patients with schizophrenia were somnolence (17%), extrapyramidal symptoms (14%), akathisia (13%), nausea (10%), and insomnia (10%).^29^ There were no suicide attempts or completed suicides during the studies included in this analysis. Suicidal ideation was observed in 4 patients receiving lurasidone (2 patients on 40 mg/d, 1 patient on 80 mg/d, 1 patient on 120 mg/d) and 3 patients receiving placebo.

## Discussion

Depression is common in patients with schizophrenia and can have considerable debilitating effects. The presence of depressive symptoms in patients with schizophrenia is associated with decreased work performance and increased risk of unemployment, decreased satisfaction with non-work activities, reduced social functioning, decreased quality of life, and increased utilization of psychiatric services.^5^ ^,^ ^10^ ^–^ ^12^ The persistence or worsening of depressive symptoms has also been associated with a significantly higher risk of psychotic relapse in patients with schizophrenia.^5^ ^,^ ^13^ ^,^ ^14^ Suicide risk is markedly elevated in patients with schizophrenia compared with the general population, and the presence of depressive symptoms contributes to this increased risk.^12^ ^,^ ^30^ ^–^ ^33^ The negative consequences associated with depressive symptomatology suggest that adequate management of these symptoms in patients with schizophrenia may reduce individual suffering, improve functioning, and contribute to reduction of the economic burden of illness.^5^

In this post hoc analysis, effects on depressive symptoms in patients with schizophrenia were analyzed utilizing MADRS assessments from four randomized, double-blind, placebo-controlled, short-term studies of lurasidone. At baseline, 45% of patients had at least mild depressive symptoms (MADRS total score ≥12); 24% of patients had depressive symptoms of at least mild to moderate severity (MADRS total score ≥16). Improvement in depressive symptoms, as measured by mean change in MADRS total score, was significantly greater at endpoint (LOCF) in the overall group of patients treated with lurasidone (N = 898) compared with those who received placebo (N = 432), and in every patient subgroup, as defined by increasing levels of baseline severity (MADRS total score ≥12, ≥14, ≥16, ≥18).

Although the effect sizes for improvement in depressive symptoms associated with lurasidone treatment in this pooled analysis were generally modest (0.25–0.34; NNTs for MADRS response and remission ranged from 11 to 14), the magnitude of effect is considered to be clinically relevant, particularly in view of the relatively low level of depressive symptom severity present for the most part at baseline, which may have limited the potential for larger reductions in MADRS total scores. Patients enrolled in the studies included in this analysis were not required to have depressive symptoms at study entry, and baseline depressive symptom severity was likely further minimized by entry criteria requiring that patients did not exhibit active suicidal ideation or behavior. In terms of the individual studies, there was significant improvement in MADRS total scores in lurasidone-treated patients relative to patients receiving placebo in 2 studies.^17^ ^,^ ^20^

The average baseline MADRS total scores in the present analysis (11.5 in the lurasidone group and 11.9 in the placebo group) were somewhat lower than baseline values in studies evaluating other atypical antipsychotic agents for the treatment of depressive symptoms in patients with schizophrenia (13.3–18.0).^8^ ^,^ ^34^ ^–^ ^36^ Mean change in MADRS total score at week 6 among patients with a baseline score of ≥16 in this analysis (–9.3) was comparable to that in a similar patient subgroup of a large, multicenter study of olanzapine (–9.7),^8^ but somewhat smaller than the changes observed in an 8-week study of risperidone and olanzapine (no placebo control group) in patients experiencing significant postpsychotic depression (–14.0 and –14.1, respectively).^37^ Risperidone and quetiapine have shown significantly greater improvement compared with placebo in depressive symptoms (as measured by depressive subscales of the PANSS or Brief Psychiatric Rating Scale) in patients with schizophrenia; however, effect sizes were not reported.^38^ ^,^ ^39^ It should be noted that comparison of findings from the present analysis with previously published studies of other atypical antipsychotic agents has limitations due to differences in study design, study duration, severity of depressive symptoms at baseline, and outcome measures used.^8^ ^,^ ^34^ ^–^ ^41^

It is possible that the apparent response of depressive symptoms to antipsychotic pharmacotherapy may be a secondary effect, driven by improvement in other symptoms of schizophrenia.^42^ ^,^ ^43^ Thus, evidence for a direct effect of antipsychotic agents on depressive symptomatology requires that these factors be excluded as the source of observed changes. In the present analysis, there was a significant correlation between change in PANSS total score and change in MADRS total score, indicating that some of the reduction in depressive symptoms may be related to the improvement in other symptoms of schizophrenia. However, the majority of the variance in MADRS total score change was independent of the change in PANSS total score. Thus, reduction in patients’ depressive symptoms was, in large part, not mediated by improvement in overall psychopathology, which suggests a primary effect of lurasidone on depressive symptoms in patients with schizophrenia. This effect is consistent with the results of recent lurasidone clinical trials in patients with bipolar depression.^24^ ^,^ ^25^ However, it is notable that lurasidone produced somewhat larger treatment effects (assessed by improvement in MADRS total score) in patients with bipolar depression (effect size of 0.51 for lurasidone monotherapy relative to placebo)^24^ compared with patients with schizophrenia (0.25–0.34 in this analysis). Although the reasons for these differences are unclear, it may be that patients with schizophrenia experience a lower severity of depressive symptoms or a form of depression that is distinct from that associated with mood disorders.

Limitations of this pooled analysis include the relatively limited overall severity of depressive symptoms at study baseline, the post hoc nature of the analysis, and the lack of adjustment of nominal *P* values for multiple comparisons. Despite these limitations, the results of this post hoc analysis support the efficacy of lurasidone for depressive symptoms in patients with schizophrenia.

## Conclusion

The results of this pooled analysis indicate that lurasidone was associated with improvement in depressive symptoms, regardless of baseline severity, as assessed by the MADRS, in patients with schizophrenia. Lurasidone may provide a useful therapeutic option for patients with depressive symptoms associated with schizophrenia. Further investigation in patients with schizophrenia and depression is warranted to confirm these findings.

## Disclosures

These studies were sponsored by Dainippon Sumitomo Pharma Co., Ltd., Osaka, Japan, and Sunovion Pharmaceuticals Inc., Marlborough, MA, USA, a U.S. subsidiary of Dainippon Sumitomo Pharma Co., Ltd.

Henry A. Nasrallah has been a consultant for Boehringer Ingelheim, Grünenthal USA Inc., Janssen Pharmaceuticals Inc., Lundbeck, Merck Sharp & Dohme Corp., Novartis Corporation, Otsuka Pharmaceutical Co., Ltd., Roche/Genentech, and Sunovion Pharmaceuticals Inc.; he has served on speakers’ bureaus for Janssen Pharmaceuticals Inc., Merck Sharpe & Dohme Corp., Novartis Corporation, Otsuka Pharmaceuticals, and Sunovion Pharmaceuticals Inc.; and he has received grant/research support from Forest Pharmaceuticals Inc, Eli Lilly and Company, Otsuka Pharmaceutical Co., Ltd., and Roche/Genentech. Josephine Cucchiaro, Yongcai Mao, Andrei Pikalov, and Antony Loebel are full-time employees of Sunovion Pharmaceuticals Inc.

## Footnotes

*
Study 1 used a Brief Psychiatric Rating Scale score of ≥42, instead of the PANSS score of ≥80 criterion, and a score of ≥4 (moderate) on 2 of the 7 PANSS positive symptom subscale items, without specifying particular items.

†
Study 1 allowed use of reversible monoamine oxidase inhibitors until 1 week prior to study entry.

‡
Study 1 permitted hospital discharge at the end of week 4, unless the investigator decided that continued hospitalization was clinically indicated.
